# A PQQ-dependent oxidoreductase in *Trypanosoma cruzi* reveals a novel redox activity in a eukaryotic pathogen

**DOI:** 10.3389/fcimb.2026.1819423

**Published:** 2026-07-07

**Authors:** Juan Pablo Gallardo, Walter Jesús Lapadula, Micaela Soledad Ossowski, Gabriela Teresa Niemirowicz, Maximiliano Juri Ayub, Karina Andrea Gómez, Mariana Potenza

**Affiliations:** 1 Instituto de Investigaciones en Ingeniería Genética y Biología Molecular “Dr. Héctor N. Torres” (INGEBI-CONICET), Buenos Aires, Argentina; 2 Departamento de Fisiología, Biología Molecular y Celular, Facultad de Ciencias Exactas y Naturales, Universidad de Buenos Aires, Buenos Aires, Argentina; 3 Universidad Argentina de la Empresa (UADE), Facultad de Ingeniería y Ciencias Exactas, Buenos Aires, Argentina; 4 Instituto Multidisciplinario de Investigaciones Biológicas de San Luis (IMIBIO-SL-CONICET), Facultad de Química Bioquímica y Farmacia, Universidad Nacional de San Luis, San Luis, Argentina; 5 Instituto de Investigaciones Biotecnológicas, Consejo Nacional de Investigaciones Científicas y Técnicas (CONICET), Universidad Nacional de San Martín–Escuela de Bio y Nanotecnologías (EByN), San Martín, Argentina; 6 Facultad de Farmacia y Bioquímica, Universidad de Buenos Aires, Buenos Aires, Argentina

**Keywords:** beta-propeller domain, Chagas disease, PQQ-dependent oxidoreductase, pyrroloquinoline quinone, *Trypanosoma cruzi*, trypanosomatid metabolism

## Abstract

*Trypanosoma cruzi*, the etiological agent of Chagas disease, faces profound nutritional and redox stress throughout its life cycle, requiring exceptional metabolic plasticity for survival across insect and mammalian hosts. Despite this, several parasite-specific metabolic pathways remain poorly characterized. Pyrroloquinoline quinone (PQQ)-dependent dehydrogenases are widespread in prokaryotes, where they play crucial roles in the oxidation of various alcohols and sugars. In contrast, their presence in pathogen eukaryotes has remained uncharacterized. Here, we report that the protein Tc323 from *T. cruzi* is a PQQ-dependent oxidoreductase with conserved structural features and an expanded domain architecture. Computational structural modeling predicted that Tc323 harbors six β-propeller domains, each composed of nine blades, while phylogenetic analyses suggested that this multi-domain architecture originated before the divergence of Trypanosomatidae. Consistent with these predictions, docking simulations revealed high-affinity binding of the PQQ cofactor to all six β-propeller domains, and immunoaffinity-purified Tc323 displayed PQQ-dependent oxidoreductase activity *in vitro*. Localization studies further showed that Tc323 is a membrane-associated protein that localizes to both the endoplasmic reticulum and glycosomes throughout the parasite life cycle and is also released in extracellular vesicles. Together, these findings uncover a previously unrecognized PQQ-dependent activity in *T. cruzi* and suggest a novel redox-related enzymatic function that may contribute to parasite adaptation to nutritional and oxidative stress.

## 1 Introduction

*Trypanosoma cruzi* is a flagellated protozoan and the causative agent of Chagas disease, a neglected tropical illness that affects more than six million people worldwide, predominantly in Latin America (Frade et al., 2025). The parasite undergoes a complex life cycle involving distinct developmental stages in insect and mammalian hosts, with replicative epimastigotes in the vector midgut, infective metacyclic trypomastigotes in the hindgut, intracellular amastigotes in mammalian cells, and circulating bloodstream trypomastigotes derived from infected cells (Martín-Escolano et al., 2022). Throughout this cycle, *T. cruzi* is exposed to profound fluctuations in nutrient availability, oxygen tension, and oxidative stress, requiring marked metabolic plasticity to ensure survival and persistence (Silva et al, 2024). However, many facets of *T. cruzi* metabolism and redox biology remain elusive, hindering the identification of parasite-specific vulnerabilities (Barbosa et al., 2025). Elucidating parasite-specific enzymes is therefore essential for understanding how *T. cruzi* orchestrates redox regulation and metabolic remodeling to endure environmental fluctuations, and may reveal vulnerabilities not present in mammalian cells.

Pyrroloquinoline quinone-dependent dehydrogenases (PQQ-DHs), also known as quinoproteins, are redox enzymes that utilize the PQQ cofactor to catalyze the oxidation of alcohols and sugars (Toyama et al., 2004). In prokaryotes, PQQ-dependent alcohol dehydrogenases (PQQ-ADHs) are particularly well characterized in methylotrophic and acetic acid bacteria, where they localize to the periplasm and initiate methanol and alcohol oxidation, transferring electrons to the respiratory chain and contributing to environmental adaptation (Shinagawa et al., 1990). To date, the only experimentally validated PQQ-dependent enzyme in eukaryotes is a pyranose dehydrogenase from the basidiomycete *Coprinopsis cinerea* (CcPDH), providing the first functional evidence that PQQ can serve as a cofactor outside bacteria (Matsumura et al., 2014). In mammals, a lactate dehydrogenase from mouse fibroblasts has been shown to interact with PQQ *in vitro*; however, this enzyme is primarily NADH-dependent and lacks the structural features typical of PQQ-ADHs, such as the β-propeller fold (Akagawa et al., 2016). Additionally, a β-alanine-activating enzyme containing a PQQ dehydrogenase–like domain has been described, although its involvement in *bona fide* PQQ-dependent catalysis appears unlikely (Drozak et al., 2014).

The PQQ-ADH enzyme family exhibits structural diversity, with some members containing additional domains beyond dehydrogenase activity. Nonetheless, a defining feature common to these enzymes is the presence of a β-propeller domain composed of antiparallel β-sheets organized into repeated submotifs, or blades, which are radially arranged around a central axis. This architecture creates a hydrophobic cavity that accommodates the PQQ cofactor and a divalent metal cation—most commonly calcium—coordinated by surrounding residues (Oubrie and Dijkstra, 2000). β-Propeller domains are widespread across prokaryotic and eukaryotic proteins and can bind a variety of ligands depending on the size and chemical properties of the central cavity (Rozeboom et al., 2015). The number of blades and overall domain organization are associated with a broad spectrum of biological functions, ranging from enzymatic catalysis to molecular scaffolding and ligand recognition (Chen et al., 2011; Kopec and Lupas, 2013; Mylemans et al., 2021).

Previously, we identified a high-molecular-weight protein in *Trypanosoma cruzi*, designated as Tc323, using phage display libraries generated from B cells of patients with chronic Chagas disease (Niborski et al., 2021; Ossowski et al., 2024a). Subsequent studies demonstrated its strong immunoreactivity, highlighting its potential as a diagnostic antigen (Ossowski et al., 2024b, 2025). Tc323 contains conserved domains homologous to bacterial PQQ-ADHs and is expressed across the major life stages of *T. cruzi*, including strains with different infectivity. Comparative phylogenetic analyses further revealed that Tc323 orthologs are restricted to *T. cruzi*, *Paratrypanosoma confusum*, and a limited group of stercorarian trypanosomes, while being absent from *Trypanosoma brucei*, *Leishmania* spp., and other closely related dixenous kinetoplastids (Ossowski et al., 2024a). This restricted distribution, together with its absence in humans, suggests that Tc323 may fulfill a parasite-specific function linked to kinetoplastid biology.

Despite its immunodiagnostic relevance, the biological function of Tc323 has remained elusive. Given its predicted homology to PQQ-dependent alcohol dehydrogenases, Tc323 emerges as a compelling target for in-depth functional and structural investigation within the framework of the parasite. Hence, we investigated the evolutionary history, structural features, subcellular localization, and enzymatic activity of Tc323 to determine whether this protein, restricted to *T. cruzi* and phylogenetically related species, represents a previously unrecognized, putative PQQ-dependent enzyme. Through an integrative approach combining *in silico* analyses with experimental validation, we provide initial evidence of PQQ-dependent redox activity in a eukaryotic pathogen, laying the groundwork for future studies aimed at uncovering novel metabolic pathways in early-branching parasitic protists.

## 2 Results

### 2.1 Ancestral multi-β-propeller architecture of Tc323 is revealed by structural and phylogenetic analyses

**Figure 1:**
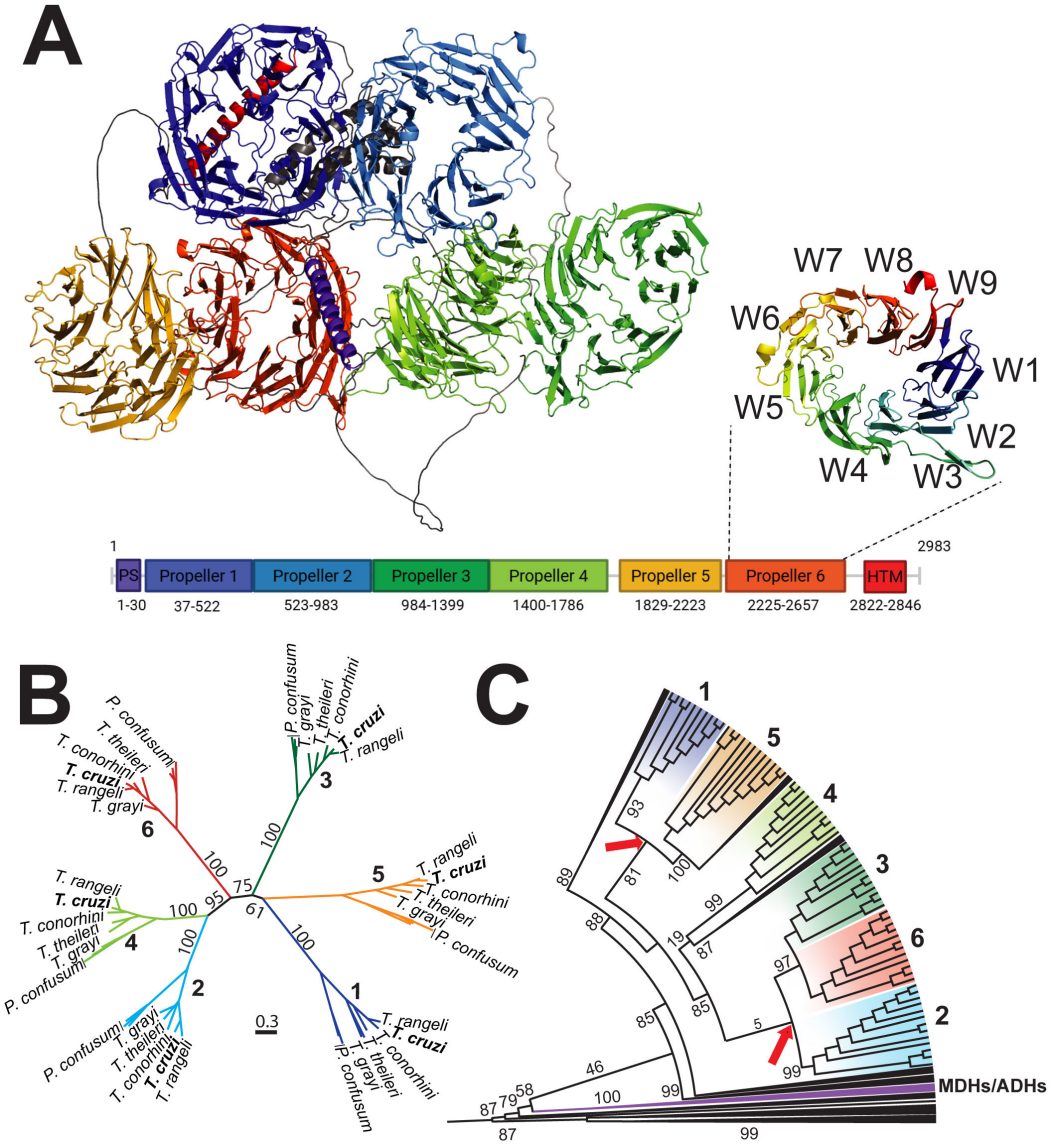
**(A)** Predicted tridimensional conformation of Tc323 (upper panel) and position of each β-propeller motif within the amino acid sequence (lower panel). β-propeller 6 is shown with its nine blades numbered (W1-W9). Confidence scores for Alpha fold prediction are shown in **Supplementary Figure 1**. PS, signal peptide. HTM, helical transmembrane motif. **(B)** Unrooted maximum-likelihood phylogeny of propellers from *Trypanosoma* and *Paratrypanosoma* species. Branches are color-coded by propeller: P1 (blue), P2 (light blue), P3 (green), P4 (light green), P5 (orange), and P6 (red). Numbers above branches indicate UFB support values for relevant nodes. **(C)** Midpoint-rooted maximum-likelihood phylogenetic tree of β-propeller domains. All clades were collapsed except those corresponding to individual propellers from *Paratrypanosoma* and *Trypanosoma* sequences, which are colored as in panel **(B)** Collapsed clades in red represent sequences with predicted ADH function, while collapsed clades in black correspond to proteins containing a β-propeller domain but associated with other enzymatic activities. Clades including sequences previously reported by Keltjens et al. (2014) are shown in violet. Red arrows indicate the nodes supporting the clustering of *Trypanosoma* and *Paratrypanosoma* sequences. A fully annotated phylogeny indicating the organismal origin of the 421 β-propeller sequences is provided in **Supplementary Figure 4**.

We previously showed that Tc323 and its homologs from phylogenetically related species exhibit a discontinuous distribution within the Trypanosomatidae family and that initial sequence-based analyses predicted a single functional domain, corresponding to a β-propeller motif related to PQQ-dependent alcohol dehydrogenases (Ossowski et al., 2024a). Here, using an updated version of AlphaFold, which supports sequences longer than 1,500 amino acids, we successfully modeled the full-length Tc323 protein and identified six putative β-propeller domains spanning the non-cytoplasmic region (Ossowski et al., 2024b; **Figure 1A** and **Supplementary Figure 1**). This analysis further predicted that all β-propeller domains adopt a nine-bladed architecture, with each blade composed of four-stranded antiparallel β-sheets (**Supplementary Figure 2**). In addition, all Tc323 homologs were found to exhibit six putative β-propeller domains (**Supplementary Figure 3**). A complementary analysis of these folds using InterPro indicated that several sequences belonging to β-propellers 2, 3, 4, and 6 were related to IPR011047, which corresponds to PQQ-dependent ADHs containing 8-bladed β-propeller domains. In the case of propeller 1, the sequence from *Trypanosoma conorhini* was associated with IPR011044, a 7-bladed β-propeller domain, suggesting that each β-propeller should be considered a discrete evolutionary unit. Therefore, we performed a phylogenetic analysis including the six β-propellers from *Paratrypanosoma* and *Trypanosoma* genus, and the resulting tree showed that each domain forms a distinct clade, with *Paratrypanosoma* sequences branching basally (**Figure 1B**). This pattern is consistent with the existence of an ancestral six-domain protein before the divergence of the trypanosomatid lineage, approximately 200 million years ago (MYA, Kaufer et al., 2019).

Given that the β-propeller fold is present across diverse protein families, we sought to further explore the evolutionary relationships of Tc323 with other sequences carrying the IPR011047 domain. By homology searches, we compiled a dataset of 461 domain sequences (**Supplementary Table ST1**). A multiple sequence alignment was generated as described in the Methods, and a maximum-likelihood phylogenetic tree was inferred (**Figure 1C**, **Supplementary Figure 4**). The resulting topology shows that trypanosomatid β-propeller domains are not monophyletic. Instead, they segregate into two sister clades (highlighted with red arrows), both also comprising domains from uncharacterized proteins of plant and fungal lineages. The first clade includes trypanosomatid domains 1 and 5 (Ultrafast Bootstrap, UFB: 81), whereas the second encompasses domains 2, 3, 4, and 6 (UFB: 85). These relationships suggest that, before the divergence of the lineages leading to plants/fungi/metazoa and trypanosomatids (approximately 1,600 MYA; http://www.timetree.org/), at least two ancestral domains were already present, which later expanded into the six domains found in trypanosomatids. Moreover, these analyses place domains from biochemically characterized ADH and MDH enzymes outside the predicted clades, revealing a discrepancy between TriTrypDB-based domain assignments and phylogenetic inference for Tc323.

### 2.2 Tc323 is a membrane-associated protein

**Figure 2:**
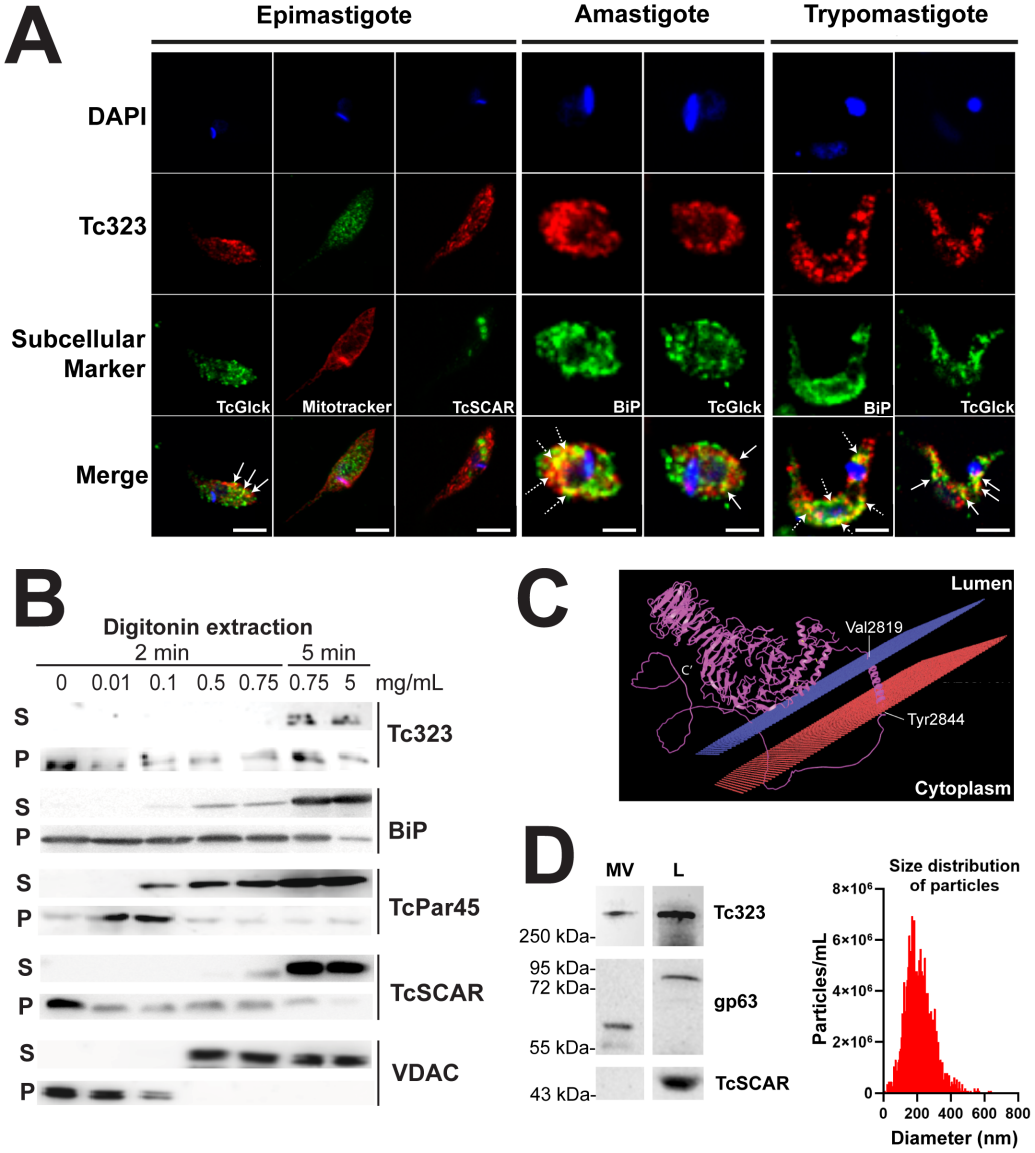
**(A)** Analysis of Tc323 co-localization with subcellular markers by immunofluorescence microscopy in the different stages of *T. cruzi*. Arrows indicate regions of co-localization. Scale bars: 1 µm for amastigotes, 2 µm for trypomastigotes, 3 µm for epimastigotes. **(B)** Western blot analysis of digitonin-extracted samples. Each lane was loaded with protein extracts from 4 × 10^6^ epimastigote cells. Samples consisted of equal protein amounts of supernatant (S) and pellet (P) fractions obtained from extractions performed with increasing digitonin concentrations (0–5 mg/mL). TcGlcK, TcSCAR, BiP, VDAC, and TcPar45 correspond to signals generated by antibodies against glucokinase, serine carboxypeptidase, endoplasmic reticulum-resident chaperone, voltage-dependent anion channel 1, and parvulin 45, respectively. **(C)** Predicted topology of the Tc323 C-terminal domain and its putative orientation within a biological membrane. C′ denotes the C-terminal end. **(D)** Left panel: Western blot analysis of microvesicles derived from *Trypanosoma cruzi*. L, total *T. cruzi* protein extract; MV, *T. cruzi*-derived microvesicles. Right panel: Particle size distribution profile of vesicles isolated by ultracentrifugation from *T. cruzi* culture supernatants.

In our previous work, we demonstrated that Tc323 localizes intracellularly in the main developmental forms of *T. cruzi*, displaying a membrane-associated pattern (Ossowski et al., 2024a). To further define its subcellular localization, we performed immunofluorescence microscopy using the anti-Tc323 antibody (chim m6B6) in combination with markers of different subcellular structures. Co-staining with an antibody against the endoplasmic reticulum (ER)-resident chaperone (BiP) antibody revealed different degrees of co-localization patterns with Tc323 for the developmental stages analyzed (dotted arrows in **Figure 2A**), with a Pearson’s correlation coefficient (PCC) of 0.61 ± 0.08, 0.59 ± 0.05 and 0.74 ± 0.05 for amastigotes, trypomastigotes and epimastigotes, respectively. In contrast, an antibody against the glycosomal enzyme Glucokinase (TcGlcK) showed lower co-localization with Tc323 in these stages (line arrows in **Figure 2A**), with PCC values of 0.42 ± 0.07, and 0.45 ± 0.06 for amastigotes, and trypomastigotes, respectively. In epimastigotes, a PCC value of 0.61 ± 0.16 was observed in epimastigotes, aligning with our previous finding that Tc323 is not exclusively ER-localized in this stage (Ossowski et al., 2024a). Notably, the localization pattern of Tc323 did not overlap with that of mitochondria or reservosomes in epimastigotes (**Figure 2A**). Taken together, these results indicate that Tc323 exhibits dual localization to the ER and glycosomes across major developmental stages.

To further assess the association of Tc323 with intracellular membranes, we performed digitonin extraction assays followed by Western blot analysis using protein lysates from epimastigotes treated with increasing concentrations of the zwitterionic detergent (**Figure 2B**). Probing with the chim m6B6 antibody revealed that low to moderate detergent concentrations did not release Tc323 into the extracts, in contrast to proteins localized to the lumen of specific organelles (the serine carboxypeptidase TcSCAR, TcGlcK, BiP, or the peptidyl-prolyl isomerase parvulin45, TcPar45). The same result was obtained using an antibody against the mitochondrial outer membrane protein voltage-dependent anion channel 1 (VDAC), a β-barrel channel protein containing multiple transmembrane β-strands that forms a pore in the outer mitochondrial membrane of *T. cruzi* (Bustos et al., 2015). In contrast, higher digitonin concentrations were required to detect Tc323 in the supernatant, suggesting a strong membrane association characteristic of integral membrane proteins, as reported previously (Schoijet et al., 2020). Accordingly, this evidence is consistent with both the InterPro analysis, which predicts association of Tc323 with the cellular membrane near its C-terminus (**Figure 1A**, lower panel), and our simulations (**Figure 2C**), which predict an alpha-helix spanning the biological membrane, comprising residues Val2819 to Tyr2844.

Finally, Tc323 was detected in extracellular vesicles isolated from epimastigote cultures by Western blot analysis (**Figure 2D**, left panel). An anti-gp63 antibody against the EV-associated surface metalloprotease gp63 was used as a positive control for the microvesicle preparation. As a control for contamination with intracellular material, antibodies against the lysosomal/reservosomal TcSCAR confirmed its absence in the vesicle preparation. Notably, nanoparticle tracking analysis (NTA) demonstrated that the preparation consisted of particles within the expected size range for microvesicles (Fernandez-Becerra et al., 2023), and no detectable whole cells or cellular debris were observed (**Figure 2D**, right panel).

### 2.3 Tc323 exhibits PQQ-dependent activity

**Figure 3:**
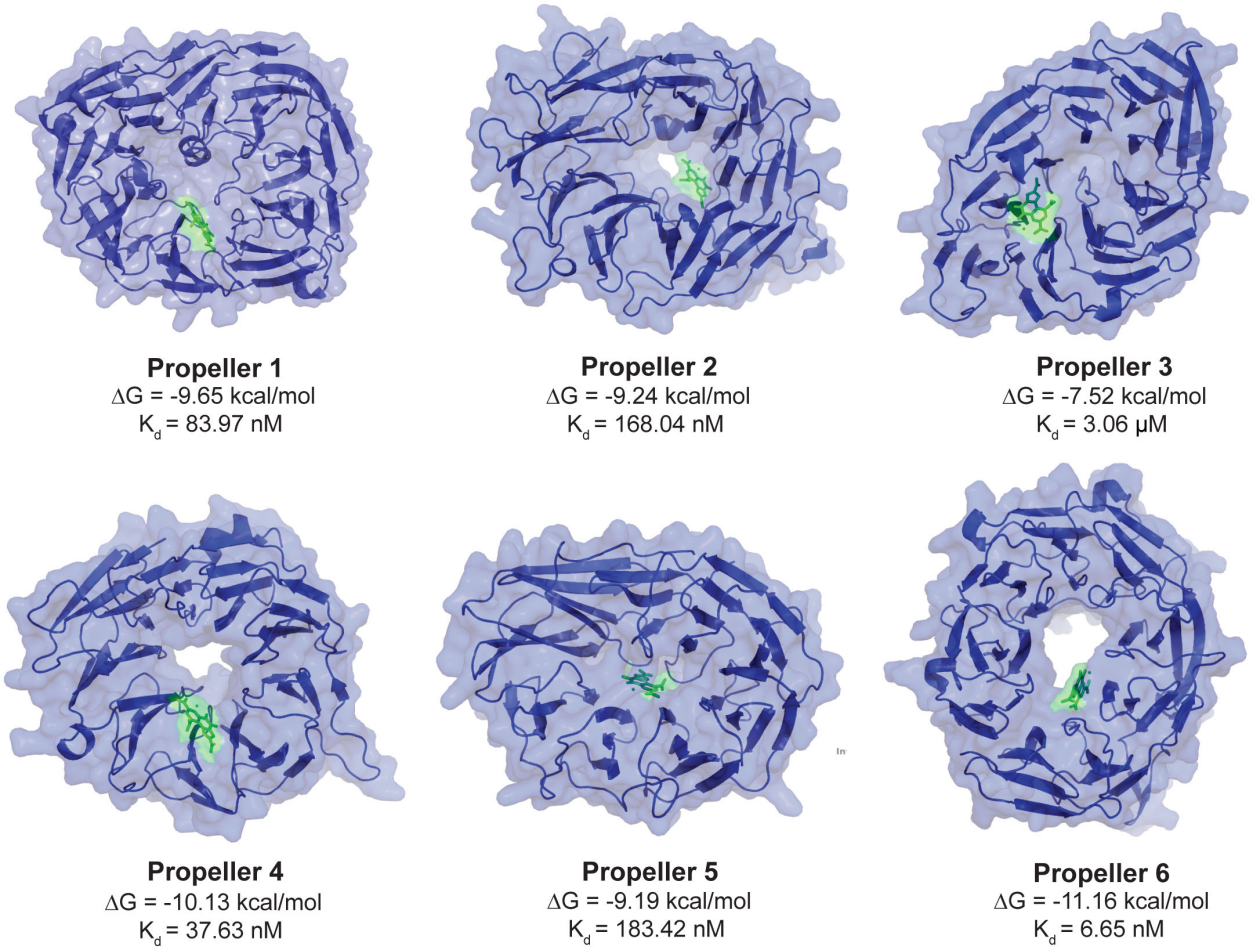
Molecular docking of the β-propeller motifs with calcium and PQQ. Ribbon blue diagrams represent the most stable conformation for each propeller. Inside each pocket, the PQQ molecule is shown in green as a stick representation.

Based on our finding that Tc323 localizes to organelles essential for parasite metabolism and the presence of several putative β-propeller domains in its sequence, we sought to determine whether PQQ indeed acts as a cofactor for Tc323. First, we analyzed the potential interaction between each putative β-propeller motif and the PQQ cofactor *in silico*. By performing molecular docking simulations, we found that PQQ bound within the individual pockets formed by each of the six β-propeller domains of Tc323 (**Figure 3**). The predicted binding free energies (ΔG°) were all below –7.52 kcal/mol, with dissociation constants (K_d_) under 3.06 μM. Notably, β-propeller 6 showed the strongest predicted binding affinity, with a ΔG° of –11.16 kcal/mol and a K_d_ of 6.65 nM. PQQ could interact with specific acidic residues within these pockets: β-propellers 1, 5, and 6 engaged the cofactor through aspartic acid residues (Asp209, Asp1976, and Asp2328, respectively), while β-propellers 2, 3, and 4 form interactions with glutamic acid residues (Glu975, Glu1377, and Glu1494). The deprotonated forms of aspartic or glutamic acid (aspartate or glutamate, respectively) interacted with both the Ca^2+^ ion and the cofactor, which in turn could act as a base catalyst for substrate oxidation. Interestingly, β-propeller 4 contains one tryptophan residue (Trp1508) close to the docked PQQ that could help position the cofactor for catalysis, due to the interaction between the rings present in the residue side chain and in PQQ. However, no pair of adjacent cysteines was found in any of the six β-propellers of Tc323, which in some PQQ-ADHs contribute to cofactor positioning. Instead, other residues stabilize its position, as seen in the protein–ligand complex diagrams (**Supplementary Figure 5**).

**Figure 4:**
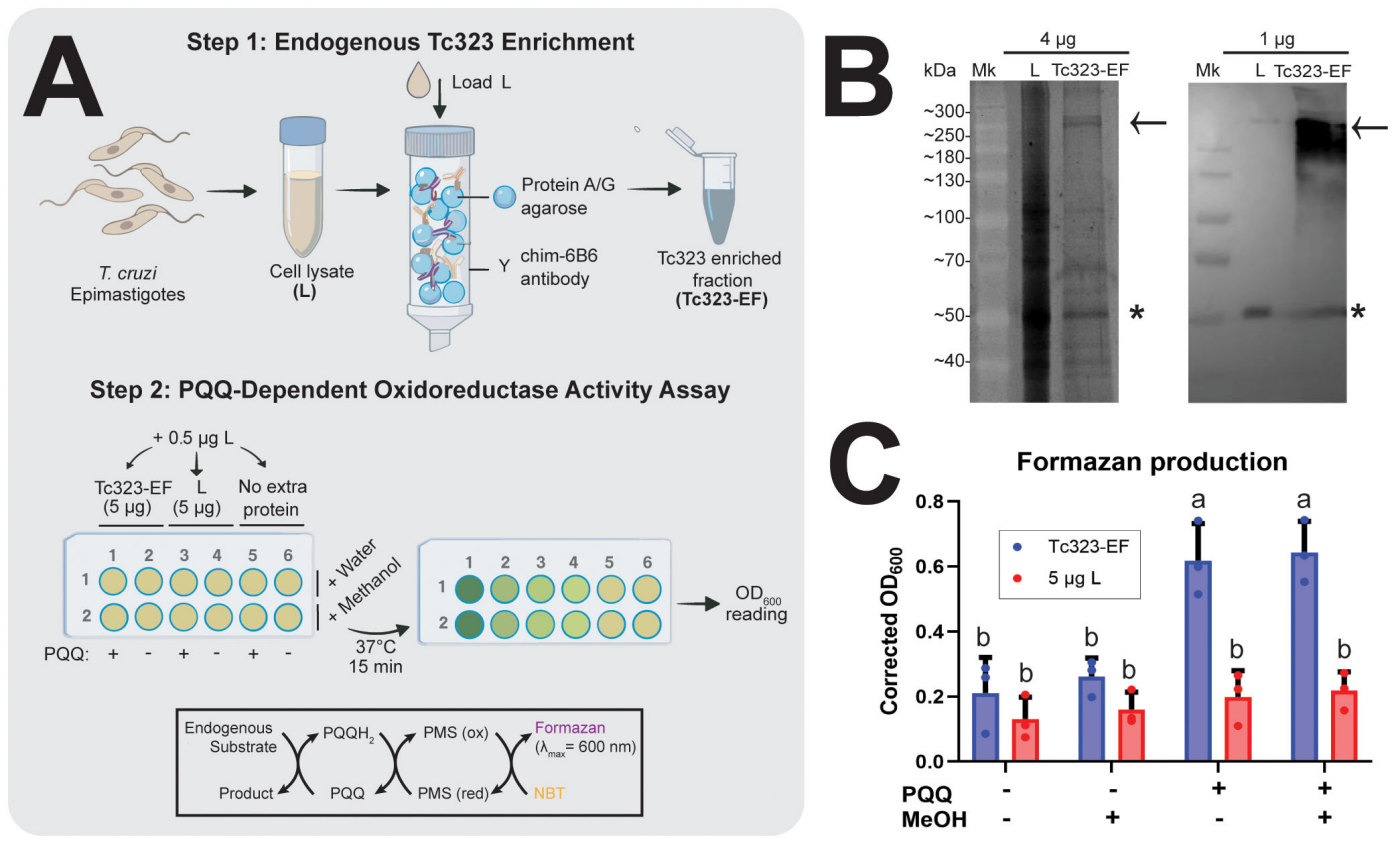
**(A)** Schematic representation of the workflow used for the endpoint NBT reduction assay. In step 1 (upper panel), the total *T. cruzi* protein lysates (L) were subjected to immunoaffinity chromatography to obtain Tc323-enriched fractions (Tc323-EF). In step 2 (lower panel), Tc323-EF supplemented with lysate (L), as well as L alone, were then incubated with nitro blue tetrazolium (NBT) in the presence or absence of PQQ and methanol. The inset box at the bottom represents the proposed electron transfer pathway, in which Tc323-dependent oxidoreductase activity, stimulated by PQQ, promotes electron transfer to NBT, generating reduced formazan, which was quantified by absorbance at 600 nm (OD600). Ox: oxidized; Red: reduced. **(B)** Gel electrophoresis and Western blot of endogenous Tc323 immunoprecipitated from *T. cruzi* protein lysates, stained with SPYRO Ruby or detected using the chim 6B6 antibody, respectively. The amount of protein loaded per lane is indicated above each panel in parentheses. kDa, molecular weight marker in kilodaltons. **(C)** Tc323 enhances PQQ-dependent NBT reduction in *T. cruzi* epimastigote extracts. Reactions containing Tc323-EF in the presence of PQQ showed significantly higher NBT reduction compared to all other conditions (two-way ANOVA with Tukey’s multiple comparisons test, p < 0.05). No significant differences were observed between Tc323-EF samples containing methanol and those containing water. NBT reduction was quantified as formazan formation (OD600) after 15 min in reactions containing Tc323-EF or total epimastigote extract (control), in the presence or absence of PQQ as indicated. Data were background-corrected against baseline reactions lacking added protein fractions. Bars represent mean ± SD (n = 3). Different lowercase letters indicate statistically significant differences among groups (*p* < 0.05).

Next, we investigated whether PQQ can function as a cofactor for Tc323 *in vitro*. To do this, Tc323-enriched fractions (Tc323-EF) were obtained from *T. cruzi* epimastigote lysates (L) by immunoaffinity chromatography, and their oxidoreductase activity was assayed by quantifying formazan production resulting from NBT reduction in the presence or absence of exogenous PQQ (**Figure 4A**). Polyacrylamide gel electrophoresis of Tc323-EF and L fractions revealed a specific increase in Tc323 after immunoprecipitation (**Figure 4B**, left panel). Hence, the densitometric analysis of Western blots normalized to tubulin showed comparable levels between the input and immunoprecipitated fractions and an approximately 5-fold augmentation in Tc323. (**Figure 4B**, right panel). Reactions were performed using Tc323-EF or an equivalent amount of total L extract as a control. Consistent with PQQ-dependent activity, Tc323-EF fractions reduced NBT more efficiently in the presence of PQQ, with cofactor addition increasing the activity to 243.45% ± 36.65%. This increase was independent of methanol supplementation, as inclusion of this alcohol did not further enhance formazan production under these experimental conditions. In contrast, PQQ did not significantly affect activity in samples containing total *T. cruzi* protein extract alone, indicating that the cofactor-dependent increase is specific to Tc323 (**Figure 4C** and Data Sheet DS1).

Together, these enrichment-based findings provide evidence that Tc323 is a PQQ-dependent oxidoreductase in *T. cruzi* and represent the first report of such activity in a pathogenic eukaryote.

## 3 Discussion

Previously, we reported that Tc323 is recognized by serum samples from patients with chronic Chagas disease, supporting its potential as a diagnostic marker, and demonstrated that this kinetoplastid-restricted protein is expressed across multiple developmental stages and strains of *T. cruzi* (Ossowski et al., 2024a, b; Ossowski et al., 2025). In the present study, we show that Tc323 comprises six β-propeller domains corresponding to InterPro entry IPR011047 and exhibits *in vitro* oxidoreductase activity that depends on PQQ as a redox cofactor, representing, to our knowledge, the first demonstration of PQQ-dependent enzymatic activity in a trypanosomatid.

Here, we employed a sequence-based search strategy, which revealed that the β-propeller domains of Tc323 likely originated from at least two ancestral domains already present in the common ancestor of plants, fungi, metazoans, and kinetoplastids. Our analyses support the vertical inheritance of these domains, with the absence of close homologs in animals likely resulting from lineage-specific gene loss—a common evolutionary mechanism affecting (Kuraku, 2010). Similar gene loss events are evident in the evolutionary history of this six-domain protein within kinetoplastids (Kuraku, 2010). Interestingly, in *T. conorhini*, two open reading frames corresponding to this protein (XP_029226016 and XP_029226017) were identified, likely resulting from a premature stop codon and a frameshift mutation near the end of β-propeller 3, consistent with ongoing pseudogenization or gene loss. Such lineage-specific patterns of retention or loss of metabolic enzymes may reflect differential selective pressures associated with adaptation to free-living versus parasitic lifestyles.

The InterPro entry IPR011047 defines the superfamily of PQQ-dependent alcohol dehydrogenases (also named quinoprotein alcohol dehydrogenases). These enzymes were originally described in bacteria and archaea, where they play central roles in alcohol and aldehyde oxidation linked to respiratory electron transport (Goodwin and Anthony, 1998; Matsushita et al., 1994). Although Tc323 shares evolutionary ancestry with prokaryotic PQQ-ADHs, its β-propeller domains are phylogenetically closer to plant and fungal sequences with similar architectures. Several of these eukaryotic homologs are annotated as putative alcohol dehydrogenases, suggesting partial conservation of structural features, although functional equivalence cannot be inferred in the absence of biochemical validation.

Structural modeling predicted that Tc323 adopts an unusual architecture consisting of six nine-bladed β-propeller domains. This organization differs from most characterized PQQ-dependent dehydrogenases, which typically contain one to three β-propeller domains with six- or eight-bladed folds (Rozeboom et al., 2015; Cao et al., 2018). Notably, the only other eukaryotic PQQ-dependent enzyme with demonstrated activity, the pyranose dehydrogenase CcPDH from *Coprinopsis cinerea*, exhibits a six-bladed β-propeller fold coordinating PQQ like bacterial enzymes (Takeda et al., 2019, 2020). In contrast, nine-bladed β-propeller domains in eukaryotes are most commonly associated with protein–protein interactions rather than catalytic activity (Liu et al., 2014), highlighting the unusual nature of Tc323 within this structural family. While AlphaFold3-based modeling provides a useful structural framework for Tc323, ongoing structural and biochemical studies are expected to help validate the predicted organization of the protein. Consistent with the predicted molecular weight, Western blot analyses using anti-Tc323 antibodies detected a protein band of approximately 323 kDa (Ossowski et al., 2024a). However, further structural validation was not feasible, as recombinant expression of Tc323 proved challenging under the conditions tested.

In our analysis, domain annotation analyses revealed heterogeneity among the six β-propeller modules of Tc323. While several domains were consistently assigned to PQQ-dependent dehydrogenase signatures, others showed weaker or divergent annotations, likely reflecting substantial sequence divergence and the limited representation of nine-bladed β-propeller enzymes in current structural databases. Another remarkable feature of Tc323 is the number of β-propeller motifs, which, being six, does not resemble any other PQQ-dependent enzyme, as the vast majority of them typically possess only one to three domains (Gvozdev et al., 2012). Although in silico docking supports the possibility of PQQ binding to each domain, it is plausible that some domains contribute to structural stability, substrate accessibility, or regulatory functions rather than direct catalysis. Further experimental work will be required to determine the functional contribution of each domain.

To extend our investigation beyond its *in silico* analysis, we carried out enzymatic activity assays and found that Tc323 is capable of oxidizing an as-yet-unidentified substrate in the presence of PQQ and Ca²^+^, under the experimental conditions tested. Although physiological substrates remain to be identified, this represents the first evidence of oxidoreductase activity associated with a putative nine-bladed β-propeller. While the observed increase in NBT reduction supports a redox-active role for Tc323, this determination of kinetic parameters (Km, Vmax, kcat) for PQQ and potential substrates will be required to define its enzymatic properties.

While alcohol dehydrogenases are often characterized by substrate promiscuity (Rozeboom et al., 2015; Zhu et al., 2024), specificity in PQQ-dependent enzymes is primarily dictated by the structural and chemical features of the central cavity formed by the β-propeller blades (Igarashi et al., 1999, 2004; Takeda et al., 2019). Comparative structural analysis indicates that Tc323 retains conserved acidic residues essential for metal coordination and PQQ binding. However, certain canonical features observed in bacterial PQQ-ADHs —such as coplanar tryptophan residues and vicinal disulfide bridges— are absent in Tc323, suggesting structural divergence and potential differences in substrate recognition or specificity (Chan et al., 2021). Assuming that Tc323 functions as a PQQ-dependent enzyme *in vivo*, the origin and availability of this cofactor in *T. cruzi* remain unclear, as no biosynthetic pathway or specific transporter has been identified, and whether PQQ is acquired from environmental or host-derived sources requires further investigation. In bacteria, PQQ is either synthesized *de novo* or imported via specific transport systems (Goosen et al., 1992; Velterop et al., 1995; Puehringer et al., 2008; Gao et al., 2023; Anthony, 2001; Hantke and Friz, 2022). In contrast, neither a PQQ biosynthetic pathway nor a specific transporter has been identified in eukaryotes. In *T. cruzi*, uptake of small redox-active molecules may occur through non-specific mechanisms such as fluid-phase or adsorptive endocytosis, or via low-specificity anion transporters (Murta et al., 2001; Shahi et al., 2002). Notably, trace levels of free PQQ have been detected in mammalian tissues and plasma (Kumazawa et al., 1993; Zhu and Klinman, 2020), and PQQ-producing bacteria are present in the insect vector microbiota (Engel and Moran, 2013; Rupawate et al., 2023), suggesting that this cofactor may be accessible to the parasite in both host environments.

Proteomic and localization studies indicate that Tc323 exhibits a dynamic, stage-dependent subcellular distribution. Previous reports have identified Tc323 in reservosomes, glycosome-enriched fractions, and extracellular vesicles (Sant’Anna et al., 2009; Ulrich et al., 2011; Queiroz et al., 2014; Brossas et al., 2017). Consistent with these findings, the present study detected Tc323 in secretory microvesicles released by epimastigotes, supporting its association with extracellular vesicle populations at this developmental stage. Concurrently, immunofluorescence analyses revealed some degree of colocalization with glycosomes and the endoplasmic reticulum, but not with reservosomes. The apparent differences between proteomic and microscopy-based approaches likely reflect variations in methodological sensitivities rather than genuine biological discrepancies (Toki et al., 2017; Bao et al., 2024). Together, these results highlight the dynamic and stage-dependent subcellular distribution of Tc323, potentially linked to distinct functional roles during the parasite life cycle, although its physiological relevance remains to be elucidated. Notably, the presence of a putative N-terminal signal peptide further suggests that Tc323 may access the secretory pathway, at least at the level of the endoplasmic reticulum.

Altogether, the discovery of a large PQQ-dependent enzyme in *T. cruzi* provides *in vitro* evidence for a previously unreported redox activity. While the physiological relevance of Tc323 remains to be elucidated, these findings expand the current view of redox-related enzymes in early-branching eukaryotes and underscore the value of fundamental biochemical studies in organisms responsible for neglected diseases.

## 4 Materials and methods

### 4.1 Analysis of β-propeller structures and transmembrane domains of Tc323

Tridimensional structure prediction of Tc323 and its domains was performed using AlphaFold 3 (Abramson et al., 2024) and I-TASSER (Zheng et al., 2025; Zhou et al., 2022; Zheng et al., 2021). Based on the structural information, each β-propeller domain was separated from all proteins homologous to Tc323 identified in Trypanosomatidae. Each set of β-propeller domains was analyzed independently in InterPro (ebi.ac.uk/interpro; Blum et al., 2025)

Molecular docking experiments were performed only with the Tc323 β-propellers from *T. cruzi*. Docking was performed following the protocol described in Kaboli et al. (2017). For this, the calcium-bound PQQ complex was retrieved from the crystallized structure of the PQQ-dependent alcohol dehydrogenase from *Pseudogluconobacter saccharoketogenenes* (PDB ID: 4CVB). Later, the target protein domain and ligand structures were prepared using AutoDockTools (Morris et al., 2009). Particularly, polar hydrogens were added, and partial charges were assigned using the Kollman method. In parallel, the ligand was prepared by adding hydrogens and assigning Gasteiger charges. Docking was carried out using AutoDock Vina (Eberhardt et al., 2021; Trott and Olson, 2010), where the grid box was positioned around the protein’s center, with dimensions set to 100 Å x 100Å x 100 Å. The docking protocol was run ten times to ensure reliability, employing the Lamarckian Genetic Algorithm (LGA), and the results were evaluated based on binding energy and the interactions between the ligand and the protein. Two-dimensional protein–ligand interaction diagrams were generated using LigPlot+ (version 2.3) (Wallace et al., 1995; Laskowski and Swindells, 2011) to analyze predicted interactions between Tc323 and pyrroloquinoline quinone (PQQ).

The interaction of Tc323 domains with biological membranes was evaluated with the TMPfold server from the Orientations of Proteins in Membranes (OPM) database (Lomize et al., 2012, 2022; opm.phar.umich.edu/).

All structures were visualized using PyMOL3.1 (https://pymol.org/).

### 4.2 Homology searches and sequence analyses

The predicted domain sequences from Tc323 homologs were used as queries in the NCBI database using BLASTp and psi-BLAST with default search parameters. Among the sequences retrieved in this search, those containing three or more β-propeller domains were excluded for further analysis, as they compromised the alignment due to differences in sequence length. The only exception was the homolog from *Trypanosoma grayi*, since it belongs to the group of kinetoplastids (Ossowski et al., 2024a). Characterized PQQ-dependent alcohol and methanol dehydrogenase (ADH and MDH) sequences were retrieved from the work performed by Keltjens et al. (2014). InterPro analysis was performed to confirm and retain only the homologous domain in all selected sequences. When the collected proteins were predicted to contain more than one multi-propeller domain, these domains were separated and treated as independent evolutionary units.

### 4.3 Phylogenetic analysis

The obtained domains were used to perform an MSA using the MAFFT online server (mafft.cbrc.jp/alignment/server/) (Kuraku et al., 2013; Katoh et al., 2018). The output was visualized, and poorly aligned regions were trimmed as a block using Trimal software in custom mode under default parameters (Capella-Gutiérrez et al., 2009). The trimmed MSA containing 461 sequences and 468 sites was used to perform phylogenetic analysis by Maximum Likelihood in IQ-TREE. The phylogenetic parameters Q.pfam+G4 were selected using ModelFinder, following tree reconstruction. To estimate the robustness of the phylogenetic inference, 1000 UBF were selected. The resulting trees were visualized with FigTree (http://tree.bio.ed.ac.uk/software/figtree/).

### 4.4 Parasite cultures

All developmental forms of *T. cruzi* were from the Dm28c strain (Berná et al., 2018). Epimastigotes were grown at 28 °C in a liver tryptose infusion medium (LIT) supplemented with 10% (*v*/*v*) heat-inactivated fetal bovine serum (FBS, Natocor, Córdoba, Argentina), 100 U/mL of penicillin, 100 mg/mL of streptomycin, and 25 µg/mL of hemin. Cell-derived trypomastigotes were obtained from the culture media of infected VERO cells (multiplicity of infection, MOI 2:1) in complete RPMI-1640 medium supplemented with 3% inactivated FBS, 100 U/mL penicillin, 100 μg/mL streptomycin, and 2 mM L-glutamine (all from Gibco, Thermo Pierce Scientific, CA, USA) at 5 days post-infection. Axenic amastigotes were obtained by incubating cell-derived trypomastigotes in RMPI-1640 pH 5.0, supplemented with 0.4% bovine serum albumin (BSA) for 24 h at 37°C.

When indicated, epimastigotes were washed with FBS-free LIT and resuspended in the same medium containing 100 mM MitoTracker Red CMXRos (Invitrogen). After incubation at 28 °C for 30 min, staining was stopped by washing with Phosphate-buffered saline (PBS).

### 4.5 Isolation and analysis of extracellular vesicles

The purification of extracellular vesicles from *T. cruzi* epimastigotes was performed in four independent biological replicates, following the methodology previously described by Fernandez-Calero et al. (2015). Briefly, *T. cruzi* cells were grown to a cell density of 9 × 10^7^ parasites/ml in 120 mL LIT supplemented with 10% (*v/v*) FBS, washed twice in PBS, and then incubated in the same volume of LIT without FBS for 2 h at 28°C. Then, parasites were removed by centrifugation at 5,000 x g for 10 min at room temperature, and the supernatant was collected and subsequently centrifuged two times at 15,000 x g for 30 min at 4°C, and one time at 100,000 x g for 1 h at 4°C. After washing once with 0.2 μm filtered PBS, vesicles were resuspended in 50 μL of the same buffer and stored at 4°C. The hydrodynamic size distribution of the purified vesicles was measured by Nanoparticle Tracking Analysis (NTA) using ZetaView PMX-230-Z TWIN in the Instituto de Investigaciones en Microbiología y Parasitología Médica (IMPaM; UBA-CONICET). The protein composition of extracellular vesicles was analyzed by Western blot as detailed below using anti-Tc323, anti-TcSCAR, and anti-gp63 as primary antibodies.

### 4.6 Immunofluorescence microscopy

Parasite cells were harvested, washed twice in PBS, adhered to poly-L-lysine-coated coverslips, fixed for 30 min with 4% (*v*/*v*) *p*-formaldehyde, and permeabilized with 0.1% (*v*/*v*) Triton X-100 for 2 min. Slides were blocked with 2% (*w*/*v*) BSA in PBS (PBS-BSA) for 30 min and then incubated 1 h with chim m6B6 (13 μg/mL), anti-TcGlcK (1:200, Cáceres et al., 2007), anti-BiP (1:10,000, Bangs et al., 1993), or anti-TcSCAR (1:500, Sant’Anna et al., 2008) antibodies. After four washes, the slides were incubated for 1 h with goat anti-rabbit (H + L) Alexa488 or goat anti-mouse Cy3 secondary antibodies (both from Invitrogen, Thermo Pierce Scientific, CA, USA), diluted 1:500. Negative control samples processed in the absence of primary antibodies were previously reported (Ossowski et al., 2024a) and are therefore not shown here, as they consistently showed negligible background signal. The slides were washed four times again and mounted with VectaShield (Vector Laboratories Inc., Newark, CA, USA) containing 300 ng/mL 4′,6-diamidino-2-phenylindole (DAPI) for nucleic acid staining. Images were acquired on a Carl Zeiss LSM 880 confocal microscope coupled to the AiryScan module with a 63x objective. This procedure was performed in five biological replicates, and colocalization was quantified by calculating Pearson’s correlation coefficient. Each deconvoluted image was analyzed using the JACoP plug-in in Fiji ImageJ software version 1.52.

Washes and reagent dilutions were done in PBS, and antibodies were diluted in PBS-BSA. All steps were performed at room temperature.

### 4.7 Cell extracts

Protein extracts from *T. cruzi* epimastigotes were prepared resuspending 4 × 10^7^ parasites in *buffer A* (25 mM Tris-HCl, 250 mM sucrose, pH 8) or *B* (5 mM MgCl_2_, 25 mM potassium acetate, 1 mM dithiothreitol (DTT), 250 mM sucrose, 1% Triton X-100, 0.1% NP40, 20 mM Tris-HCl), supplemented with 1X protease inhibitor cocktail (Roche, Germany). Each cell suspension was then subjected to five freeze-thaw cycles using liquid nitrogen and centrifuged at 14,000 × g for 15 min. Total protein concentrations in the supernatants were determined using the Bradford assay. Samples were aliquoted and stored at −80 °C until use, depending on enzymatic assays or protein purification.

All procedures were performed at 4 °C.

### 4.8 Digitonin treatment and western blot analysis

Digitonin extraction was performed as described elsewhere (Miranda et al., 2009). Briefly, 1.8 x 10^8^ epimastigote cells were washed twice and resuspended in 525 µL of *buffer* A, plus 1X protease inhibitor cocktail. Aliquots of 75 µL with 2.5 × 10^7 cells each were mixed with increasing amounts of digitonin (0–5 mg/mL) and incubated for 2–5 min. Solubilized proteins were separated from the non-solubilized cellular material by centrifugation at 13,000 x g for 2 to 5 min. Each fraction was mixed with 5 X SDS-PAGE sample buffer, resolved on 8 or 12% polyacrylamide gels, and transferred to nitrocellulose membranes (GE Healthcare, Chicago, IL, USA). Western blot was performed blocking membranes with 5% (*w*/*v*) of skimmed milk in PBS containing 0.01% (*v*/*v*) Tween 20 (PBST) and then incubated with chim m6B6 (3 μg/mL), anti-BiP (1:10,000), anti-TcGlK (1:500), anti-Par45 (1:500, Erben et al., 2010), anti-VDAC (1:400, Santa Cruz Biotechnology), or anti-TcSCAR (1:1,000) antibodies. After four washings with PBST, the membranes were incubated with goat anti-mouse IgG-HRP or goat anti-rabbit IgG-HRP (both 1:6,000, Calbiochem, USA). Protein bands were revealed using Western Lightning^®^ Plus-ECL (Perkin Elmer) chemiluminescence reagent and visualized on a GeneGenome XRQ device (Syngene).

All antibodies were diluted in 2% (*w*/*v*) skimmed milk in PBST and incubated for 1 h at room temperature.

### 4.9 Protein enrichment

The endogenous Tc323 protein was purified by immune-affinity chromatography as described before (Ossowski et al., 2024a). Briefly, protein extracts from *T. cruzi* epimastigotes were passed through a mini column containing the chim-6B6 antibody covalently coupled to Protein A/G agarose. The resin was then washed with three volumes of buffer *B*, and protein was eluted with 0.1 M glycine-HCl, pH 2.0, and immediately neutralized with 5 µL of 1 M Tris-HCl, pH 9.0, to avoid degradation of the purified Tc323. Protein concentration was determined by Bradford assay, and the identity of Tc323 was confirmed by WB using the chim m6B6 antibody. In addition, samples were analyzed by total protein staining using SYPRO Ruby Protein Gel Stain (Invitrogen) according to the manufacturer’s instructions.

### 4.10 Enzymatic assays

PQQ-dependent oxidoreductase activity was assessed essentially as described by Good et al. (2016), with the modification that nitroblue tetrazolium (NBT) reduction was quantified at a fixed endpoint following Vemuluri et al. (2017). Briefly, protein preparations consisting of 5 µg Tc323-enriched fractions (Tc323-EF), 5 µg total *T. cruzi* epimastigote extract, or water (no additional protein fraction added) were pre-incubated in the presence or absence of 6 nmol pyrroloquinoline quinone (PQQ; AK Scientific, USA) in 100 mM Tris-HCl (pH 9.0) for 10 min at room temperature. Reactions were assembled in a final volume of 180 µL containing 100 mM Tris-HCl (pH 9.0), 87 µM CaCl₂, 5 mM NBT (Sigma-Aldrich, USA), 1 mM phenazine methosulfate (PMS; Merck, Germany), and 5 mM methylamine, and, in all cases, supplemented with 0.5 µg of *T. cruzi* epimastigote protein extract. Following reaction assembly, methanol (25 mM final; 20 µL) or an equivalent volume of water (20 µL) was added as indicated. Plates were incubated at 37°C for 15 min to allow formazan formation, which was quantified by measuring absorbance at 600 nm using a VERSAmax^®^ microplate reader (Molecular Devices, USA). OD_600_ values obtained in the presence of Tc323-EF or total extract were background-corrected using the corresponding baseline reactions lacking additional protein fraction under identical substrate and cofactor conditions. Results were expressed as the difference relative to baseline, calculated as Corrected OD_600_ = OD_600_(sample) − OD_600_(blank). To evaluate the effect of methanol addition, enzymatic activity was normalized to the corresponding water control condition and expressed as Relative activity (%) = (Corrected OD_600_ MeOH/Corrected OD_600_ H2O) × 100. Statistical significance was assessed by two -way ANOVA followed by Tukey’s multiple comparisons test, and differences were considered significant at p < 0.05.

## Data Availability

The original contributions presented in the study are included in the article/supplementary material. Further inquiries can be directed to the corresponding author.

## Supplementary material

Supplementary Table 1 Sequences included in the analysis of Tc323 β-propeller evolutionary history. For characterized MDHs and ADHs, accession codes listed correspond to those reported by Keltjens et al. (2014); updated accession numbers are also provided where the original codes are now obsolete.

Supplementary Figure 1 (Left) Cartoon representation of the predicted Tc323 structural model generated by AlphaFold, colored according to the per-residue confidence score (pLDDT). The color scale ranges from low confidence (red/orange, pLDDT < 50), indicative of potentially disordered or flexible regions, to very high confidence (blue, pLDDT > 90), corresponding to well-defined structural elements. (Right) Predicted Aligned Error (PAE) heatmap of the Tc323 model. The PAE plot shows the expected positional error (in Å) between residue pairs when aligned to each other. Darker colors indicate low predicted error and high relative confidence in the spatial relationship between residues, whereas lighter colors reflect greater uncertainty.

Supplementary Figure 2 Structure prediction of each β-propeller of Tc323.

Supplementary Figure 3 Structure prediction of Tc323 homologues within Trypanosomatidae.

Supplementary Figure 4 Midpoint-rooted maximum-likelihood phylogenetic tree of Trypanosomatidae β-propeller domains. Clades corresponding to individual propellers from *Paratrypanosoma* and *Trypanosoma* sequences are shown in pink. Predicted ADHs from plant, fungal, bacterial, and archaeal lineages are shown in red. The cyan clade indicates MDHs from bacterial and archaeal sequences previously reported by Keltjens et al. (2014). Black clades represent proteins that contain a β-propeller domain but are associated with other enzymatic activities.

Supplementary Figure 5 Two-dimensional interaction diagrams generated with LigPlot illustrating the predicted binding mode of pyrroloquinoline quinone (PQQ) within each β-propeller domain of the Tc323 structural model. Hydrogen bonds between PQQ and surrounding amino acid residues are indicated by dashed green lines, with bond distances (Å) indicated. Hydrophobic contacts are represented as red spoked arcs pointing toward the ligand. Residues are labeled according to their position in the Tc323 sequence. Green arrows indicate acidic amino acids that interact with PQQ and may act as catalytic residues.

Supplementary Data Sheet 1 Raw OD600 values corresponding to NBT reduction are shown for all experimental conditions, including Tc323-enriched fractions, total T. cruzi extracts, and blank controls. Corrected OD600 values were obtained by subtracting the corresponding blank readings. Relative activity (%) was calculated by normalizing methanol-treated samples to their respective water control condition.
