# Supplementary figures and images for: A PQQ-dependent oxidoreductase in Trypanosoma cruzi reveals a novel redox activity in a eukaryotic pathogen
Source: Front Cell Infect Microbiol. 2026 Jul 7;16:1819423. doi: 10.3389/fcimb.2026.1819423 (PMC13384848; doi:10.3389/fcimb.2026.1819423)

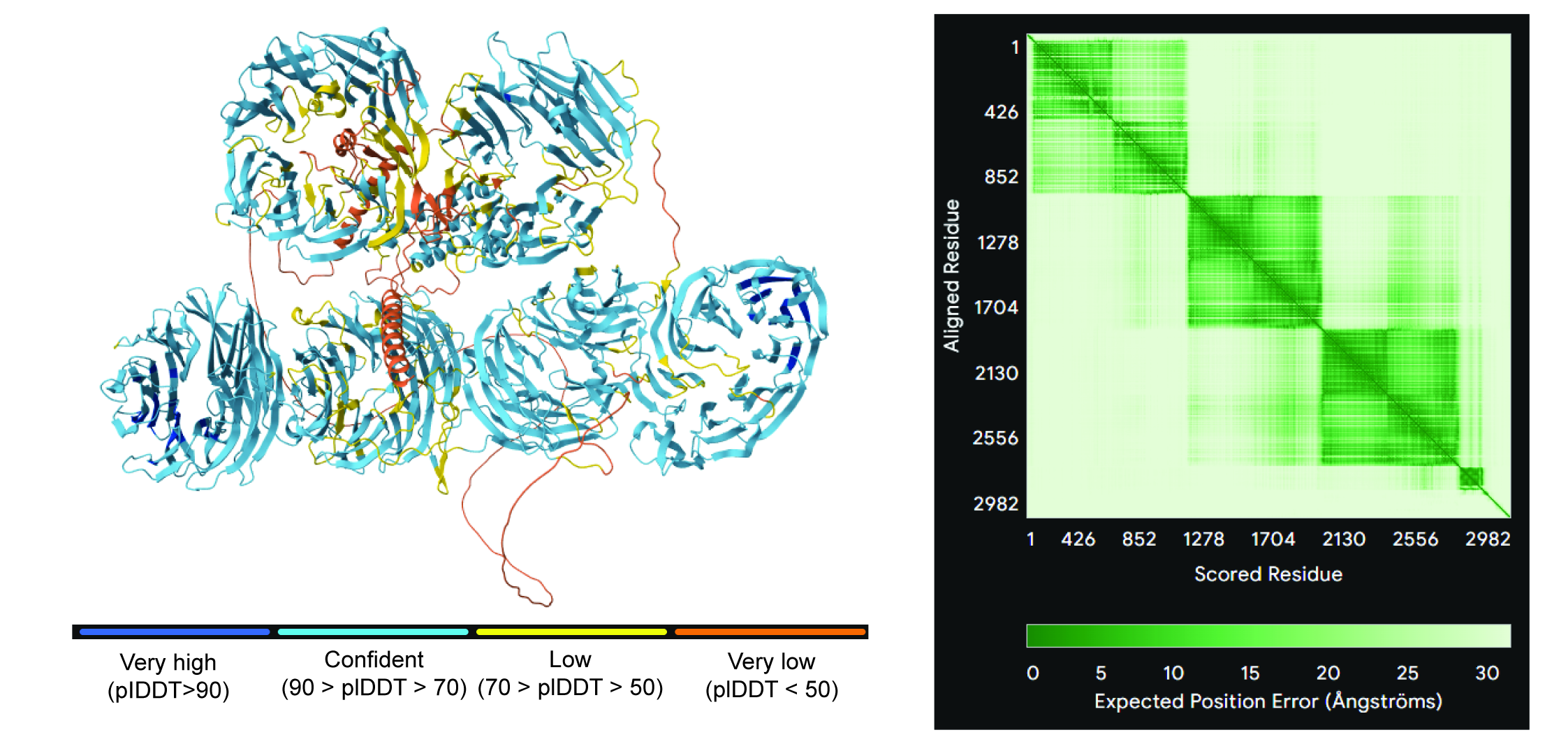

Supplement: Supplementary Figure 1 — (Left) Cartoon representation of the predicted Tc323 structural model generated by AlphaFold, colored according to the per-residue confidence score (pLDDT). The color scale ranges from low confidence (red/orange, pLDDT < 50), indicative of potentially disordered or flexible regions, to very high confidence (blue, pLDDT > 90), corresponding to well-defined structural elements. (Right) Predicted Aligned Error (PAE) heatmap of the Tc323 model. The PAE plot shows the expected positional error (in Å) between residue pairs when aligned to each other. Darker colors indicate low predicted error and high relative confidence in the spatial relationship between residues, whereas lighter colors reflect greater uncertainty. [file Image1.tif]

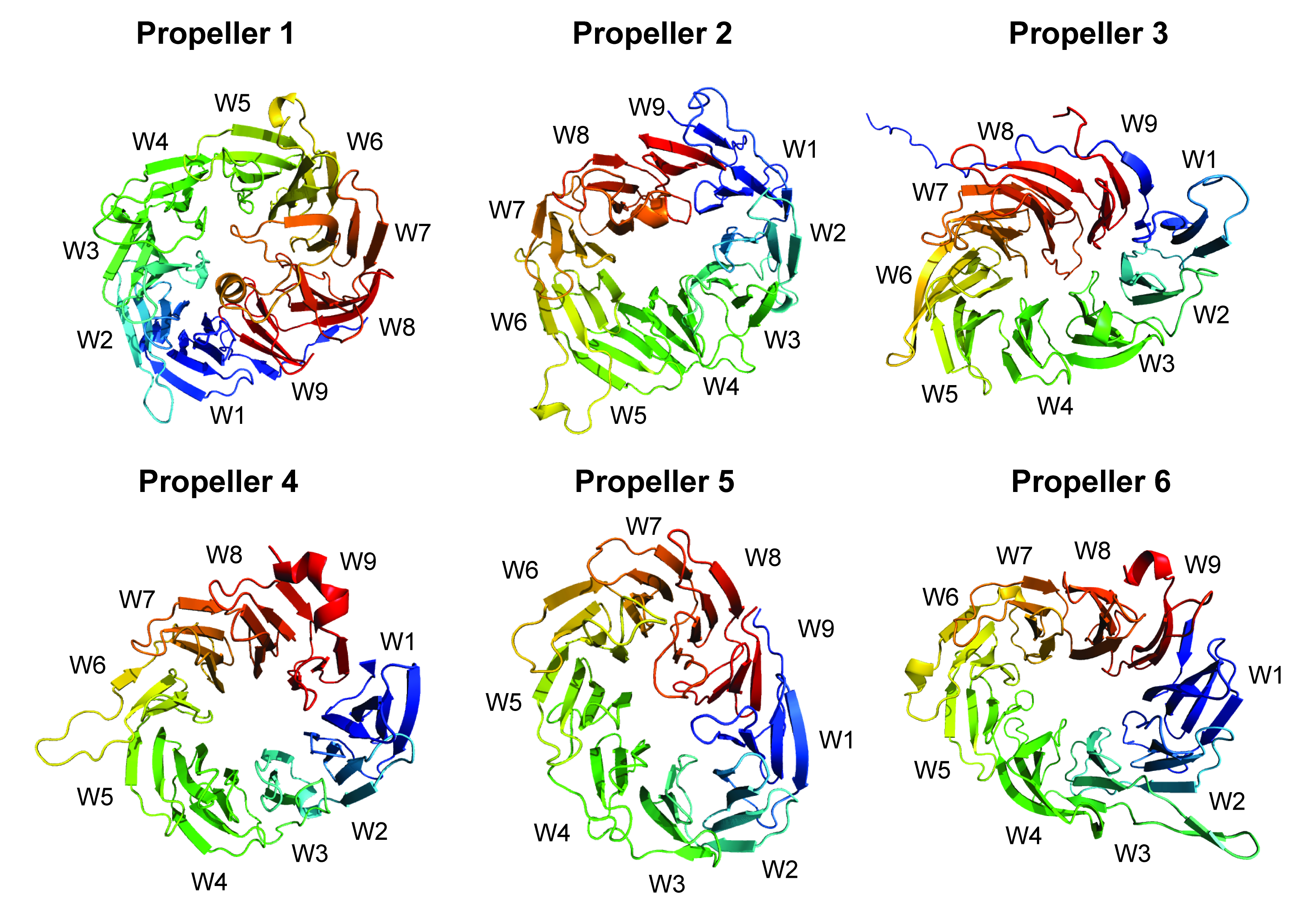

Supplement: Supplementary Figure 2 — Structure prediction of each β-propeller of Tc323. [file Image2.tif]

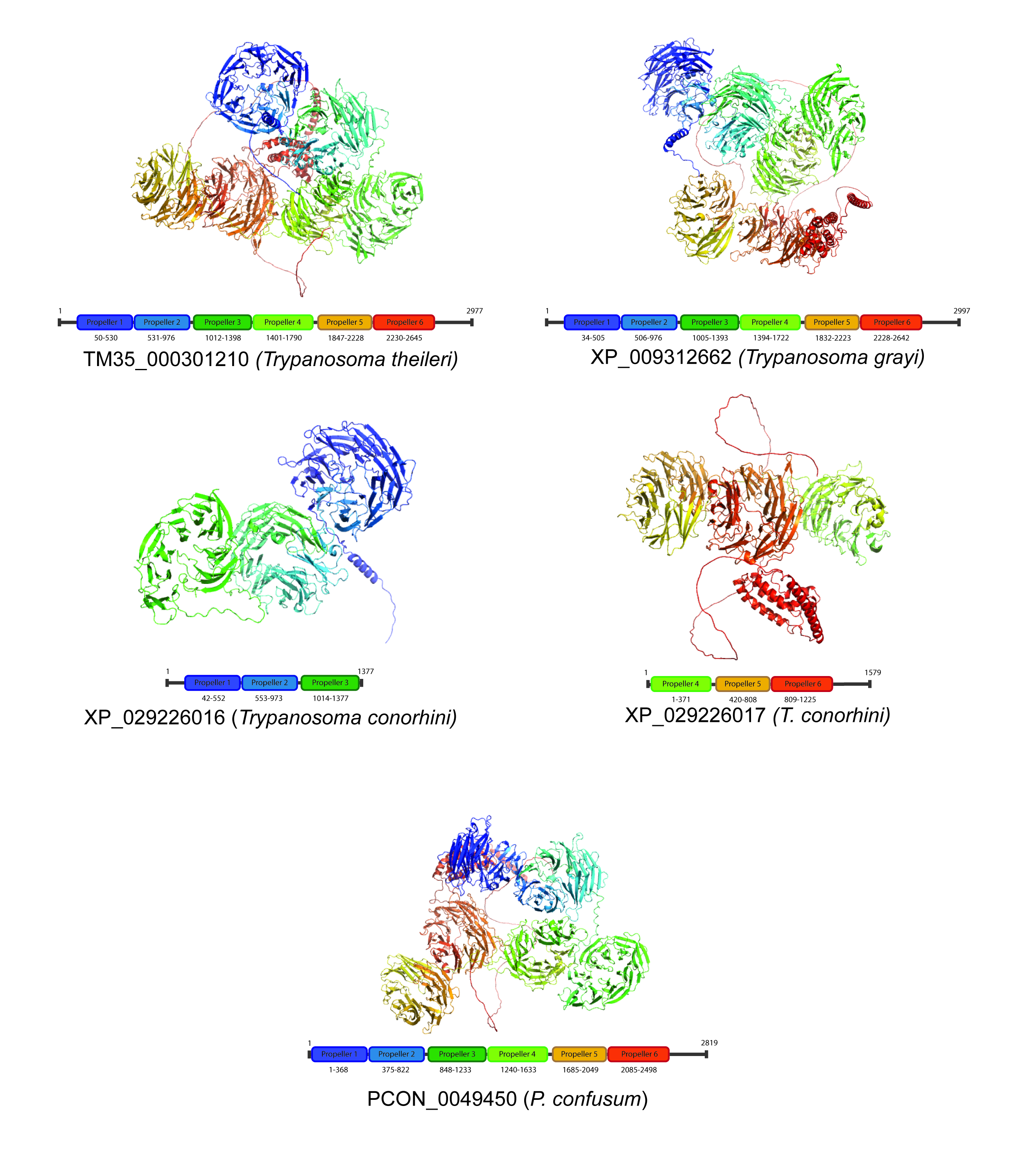

Supplement: Supplementary Figure 3 — Structure prediction of Tc323 homologues within Trypanosomatidae. [file Image3.tif]

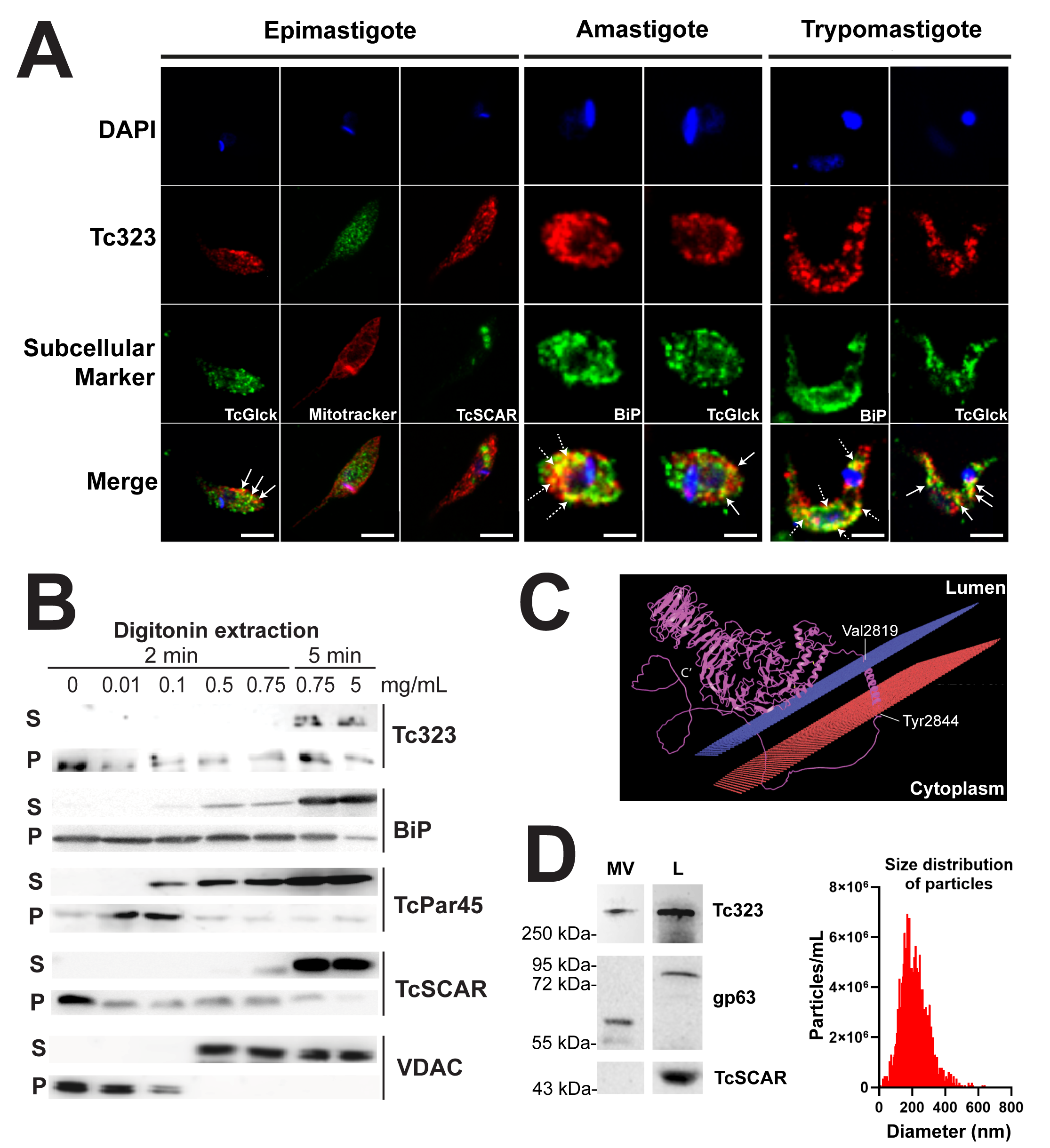

Supplement: Supplementary Figure 4 — Midpoint-rooted maximum-likelihood phylogenetic tree of Trypanosomatidae β-propeller domains. Clades corresponding to individual propellers from Paratrypanosoma and Trypanosoma sequences are shown in pink. Predicted ADHs from plant, fungal, bacterial, and archaeal lineages are shown in red. The cyan clade indicates MDHs from bacterial and archaeal sequences previously reported by Keltjens et al. (2014). Black clades represent proteins that contain a β-propeller domain but are associated with other enzymatic activities. [file Image4.tif]

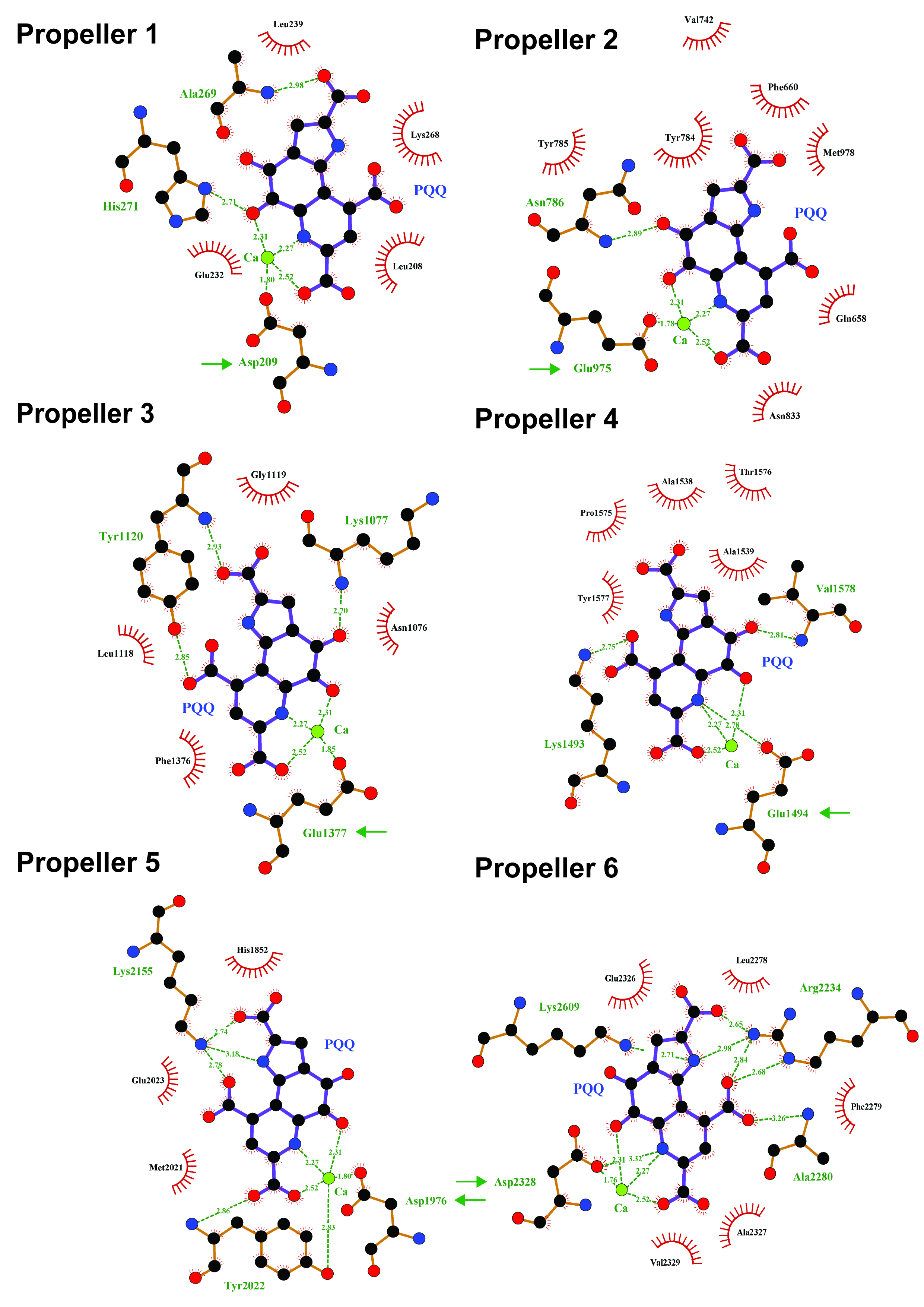

Supplement: Supplementary Figure 5 — Two-dimensional interaction diagrams generated with LigPlot illustrating the predicted binding mode of pyrroloquinoline quinone (PQQ) within each β-propeller domain of the Tc323 structural model. Hydrogen bonds between PQQ and surrounding amino acid residues are indicated by dashed green lines, with bond distances (Å) indicated. Hydrophobic contacts are represented as red spoked arcs pointing toward the ligand. Residues are labeled according to their position in the Tc323 sequence. Green arrows indicate acidic amino acids that interact with PQQ and may act as catalytic residues. [file Image5.tif]
