# Supplementary material for: A PQQ-dependent oxidoreductase in Trypanosoma cruzi reveals a novel redox activity in a eukaryotic pathogen
Source: Front Cell Infect Microbiol. 2026 Jul 7;16:1819423. doi: 10.3389/fcimb.2026.1819423 (PMC13384848; doi:10.3389/fcimb.2026.1819423)
Supplement: Supplementary Data Sheet 1 — Raw OD600 values corresponding to NBT reduction are shown for all experimental conditions, including Tc323-enriched fractions, total T. cruzi extracts, and blank controls. Corrected OD600 values were obtained by subtracting the corresponding blank readings. Relative activity (%) was calculated by normalizing methanol-treated samples to their respective water control condition. [file DataSheet1.docx]

**Supplemental material:**

OD_600_ values of formazan production due to NBT reduction in enzymatic assays:

| **Protein added** | **Substrate added** | **Added PQQ** | **OD600** | | | **Corrected OD_600_** | | | **% Relative Activity** | | |
| --- | --- | --- | --- | --- | --- | --- | --- | --- | --- | --- | --- |
|  |  |  | **Replicate 1** | **Replicate 2** | **Replicate 3** | **Replicate**  **1** | **Replicate**  **2** | **Replicate**  **3** | **Replicate**  **1** | **Replicate**  **2** | **Replicate**  **3** |
| **5 μg Tc323-EF** | **H2O** | **Yes** | 0,81 | 0,973 | 0,824 | 0,515 | 0,741 | 0,599 | - | - | - |
|  |  | **No** | 0,579 | 0,684 | 0,498 | 0,288 | 0,259 | 0,086 | - | - | - |
|  | **MeOH** | **Yes** | 0,925 | 0,962 | 0,748 | 0,634 | 0,743 | 0,553 | 123,107 | 100,270 | 92,321 |
|  |  | **No** | 0,56 | 0,708 | 0,536 | 0,281 | 0,306 | 0,198 | 97,569 | 118,147 | 230,233 |
| **5 μg *T. cruzi* Extract** | **H2O** | **Yes** | 0,518 | 0,497 | 0,335 | 0,223 | 0,265 | 0,11 | - | - | - |
|  |  | **No** | 0,403 | 0,631 | 0,487 | 0,112 | 0,206 | 0,075 | - | - | - |
|  | **MeOH** | **Yes** | 0,516 | 0,491 | 0,353 | 0,225 | 0,272 | 0,158 | 100,897 | 102,642 | 143,636 |
|  |  | **No** | 0,404 | 0,624 | 0,472 | 0,125 | 0,222 | 0,134 | 111,607 | 107,767 | 178,667 |
| **No additional protein**  **(Blank)** | **H2O** | **Yes** | 0,295 | 0,232 | 0,225 | - | - | - | - | - | - |
|  |  | **No** | 0,291 | 0,425 | 0,412 | - | - | - | - | - | - |
|  | **MeOH** | **Yes** | 0,291 | 0,219 | 0,195 | - | - | - | - | - | - |
|  |  | **No** | 0,279 | 0,402 | 0,338 | - | - | - | - | - | - |

Corrected OD_600_ calculation:

$$\text{C}\text{o}\text{rr}\text{ected}\text{ }\text{OD}_{\text{600}}\text{=}\text{OD}_{\text{600}}\left( \text{Sample} \right) \text{- }\text{OD}_{\text{600}}\left( \text{Blank} \right)$$

Percentage of activity using methanol as a substrate calculation:

$$\text{\% }\text{Fold Change in}\text{ Activity = }\frac{\text{Corrected}\text{ }\text{OD}_{\text{600}}\text{ (Sample + MeOH)}\text{×100}\text{ }}{\text{Corrected}\text{ }\text{OD}_{\text{600}}\text{ (Sample + }\text{H}_{\text{2}}\text{O)}}$$
